# Comparison of approaches to estimate confidence intervals of post-test probabilities of diagnostic test results in a nested case-control study

**DOI:** 10.1186/1471-2288-12-166

**Published:** 2012-10-31

**Authors:** Bas van Zaane, Yvonne Vergouwe, A Rogier T Donders, Karel GM Moons

**Affiliations:** 1 Julius Center for Health Sciences and Primary Care And Division of Anesthesiology, Intensive Care Care and Emergency Medicine, University Medical Center Utrecht, PO box 85500, 3508 GA, Utrecht, The Netherlands; 2 Julius Center for Health Sciences and Primary Care, University Medical Center Utrecht, PO box 85500, 3508 GA, Utrecht, The Netherlands; 3 Department of Epidemiology, Biostatistics and Health Technology Assessment, Radboud University Nijmegen Medical Center, PO Box 9101, 6500 HB, Nijmegen, The Netherlands

## Abstract

**Background:**

Nested case–control studies become increasingly popular as they can be very efficient for quantifying the diagnostic accuracy of costly or invasive tests or (bio)markers. However, they do not allow for direct estimation of the test’s predictive values or post-test probabilities, let alone for their confidence intervals (CIs). Correct estimates of the predictive values itself can easily be obtained using a simple correction by the (inverse) sampling fractions of the cases and controls. But using this correction to estimate the corresponding standard error (SE), falsely increases the number of patients that are actually studied, yielding too small CIs. We compared different approaches for estimating the SE and thus CI of predictive values or post-test probabilities of diagnostic test results in a nested case–control study.

**Methods:**

We created datasets based on a large, previously published diagnostic study on 2 different tests (D-dimer test and calf difference test) with a nested case–control design. We compared six different approaches; the approaches were: 1. the standard formula for the SE of a proportion, 2. adaptation of the standard formula with the sampling fraction, 3. A bootstrap procedure, 4. A approach, which uses the sensitivity, the specificity and the prevalence, 5. Weighted logistic regression, and 6. Approach 4 on the log odds scale. The approaches were compared with respect to coverage of the CI and CI-width.

**Results:**

The bootstrap procedure (approach 3) showed good coverage and relatively small CI widths. Approaches 4 and 6 showed some undercoverage, particularly for the D-dimer test with frequent positive results (positive results around 70%). Approaches 1, 2 and 5 showed clear overcoverage at low prevalences of 0.05 and 0.1 in the cohorts for all case–control ratios.

**Conclusion:**

The results from our study suggest that a bootstrap procedure is necessary to assess the confidence interval for the predictive values or post-test probabilities of diagnostic tests results in studies using a nested case–control design.

## Background

An essential step in the evaluation process of a (new) diagnostic test is to assess the diagnostic accuracy measures [1-4]. Traditionally the sensitivity and specificity are studied but another important measure is the predictive value, i.e. the absolute probability that the disease is present or absent given the test result, so-called post-test probability [5]. Typically, diagnostic accuracy studies use a cross-sectional design in a series or cohort of patients that is defined by the suspicion of the target disease under study. This suspicion is usually defined by the presented symptoms or signs. All patients then undergo the index (e.g. new) tests and subsequently the prevailing reference test or standard [5,6]. Subsequently the predictive values or post-test probabilities of the test results, as well as the sensitivity and specificity can be estimated.

An efficient alternative for this full cohort design is the nested case–control design, in which the controls and cases are sampled from a pre-defined cohort [5-8]. This design is particularly advantageous for diagnostic research purposes when the prevalence of the disease is rare, when the index test is costly or difficult to perform, and when using stored (e.g. biological) material from existing cohorts or biobanks [5-7,9]. Limitations, strengths and rationale of the nested case–control design are extensively discussed in the literature, mostly for etiologic research [8,10,11], but also recently for the evaluation of diagnostic tests [5,6,9].

As an important aim in diagnostic research is to estimate the absolute probability of having the disease given test results (predictive values or post-test probability), the nested character of the design in a cohort with known size is essential. In non-nested or regular case–control studies, controls are sampled from a source population with unknown size. The prevalence of the disease and hence the predictive values can thus not simply be estimated [5,6]. Only relative probabilities, like the odds ratio, can directly be estimated. However, absolute disease probabilities can be estimated, if cases and controls are sampled from an existing, pre-defined cohort, by weighing with the inverse sampling fraction [5].

**Figure 1:**
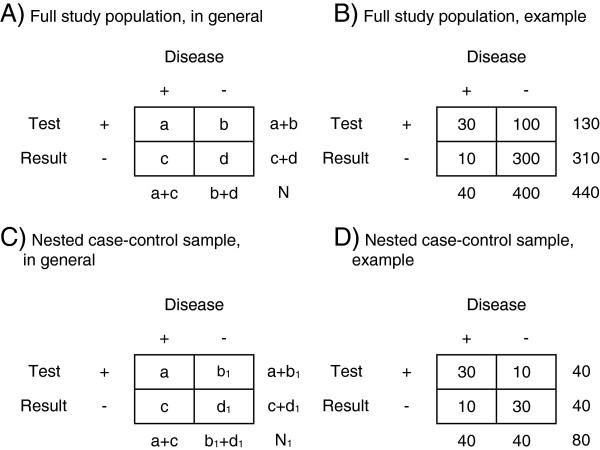
**Theoretical example of case–control sample, nested in a cohort with known size and prevalence of the disease.** (**A** = true positive, **B** = false positive, **C** = false negative, **D** = true negative, N = number of patients).

For example, consider a full-cohort approach in which the index test result and reference test results are assessed for all patients. Say the index test is an expensive dichotomous biomarker (genomic) measurement requiring human material that is frozen for all cohort members in a biobank. The positive predictive value (PPV) of the marker result is $\frac{a}{a+b}$, and the negative predictive value (NPV) $\frac{d}{c+d}$ (Figure 1, Table A, see legend of Figure 1 for explanation of variable names).

In a nested case–control design, one samples from the full cohort (commonly) the human material of all subjects with a positive reference test (cases), but only a fraction (see cell b1 and d1, Figure 1, Table C) of those with a negative reference test (controls). The expensive index test is thus only retrieved or measured in the human material of the sampled cases and controls.

In contrast to the typical case–control design, in this nested design the absolute disease probabilities can be calculated by weighing the denominator with the inverse sampling fraction: the PPV = $\frac{a}{a+\frac{1}{\text{sampling}\;\text{fraction}}\cdot b_{1}}\text{,}$ and the NPV = $\frac{d_{1}\cdot \frac{1}{\text{sampling}\;\text{fraction}}}{c+d_{1}\cdot \frac{1}{\text{sampling}\;\text{fraction}}}\text{,}$ with sampling fraction $\frac{b_{1}+d_{1}}{b+d}$ (Figure 1, Table C). For example, the PPV and NPV from the full study are 30/(30+100)=0.23 and 300/(10+300)=0.97 (Figure 1, Table B). Applying the approaches for the nested case–control sample with only 10% of all non-diseased patients yields the same results. Sampling fraction = (10+30)/(100+300)=0.1, PPV=30/(30+(10 · 10)) = 0.23, NPV = (30 · 10)/(10+(30 · 10))=0.97 (Figure 1, Table D).

However, the estimation of the standard error (SE) of the predictive values derived from a nested case–control diagnostic accuracy study is not at all straightforward. When simply using the standard formula for the SE of a proportion ($\sqrt{\frac{\pi \left(1-\pi \right)}{n}}$, where π is the proportion, here predictive value or absolute disease probability, and *n* the number of patients, the question is which value for *n* to use. The actual observed (measured) number of cases and controls does not correspond to the estimated proportion (too low). But simply using the upwardly corrected number of controls and (if also sampled) cases, falsely increases the number as if they were all observed, yielding too small SE’s. Clearly, modifications have to be made to the standard formulas, to estimate the correct SE of the predicted values of a diagnostic index test from a nested case–control study.

Recently Mercaldo and colleagues published a approach to estimate the SE of predictive values for a case–control approach [12].We compared the approach proposed by Mercaldo with five other approaches using simulated datasets based on an empirical published diagnostic study among patients suspected of deep venous thrombosis. We studied several clinically relevant combinations of disease prevalence and case–control ratios.

## Methods

### Patient data

We used data from a published cross-sectional diagnostic study that collected a cohort of 2086 adult patients suspected of deep vein thrombosis (DVT) in primary care [13,14]. In brief, the general practitioners systematically documented information on patient history and physical examination. Physical examination included swelling of the affected limb and difference in circumference of the calves calculated as the circumference (in centimeters) of affected limb minus circumference of unaffected limb, further referred to as calf difference test. The calf-difference was considered to be abnormal if the difference in circumference between the legs was more then 3 cm. Subsequently, all patients underwent D-dimer testing.

Depending on the hospital to which the patient was referred in the original study the ELISA approach (VIDAS, Biomerieux, France) or the latex assay approach (Tinaquant, Roche, Germany) was used to determine the D-dimer level. The test was considered abnormal if the latex assay yielded a D-dimer level ≥400 ng/mL (Tinaquant, Roche, Germany) or ≥500 ng/mL for the ELISA assay (VIDAS, Biomerieux, France) [15]. Values were dichotomized: normal versus abnormal. In the present approachological study, we focus on the calf difference and D-dimer test as index tests. Presence of DVT (yes/no) was assessed in all patients with the reference test (repeated compression ultrasonography of the symptomatic leg).

### Nested case–control samples

**Figure 2:**
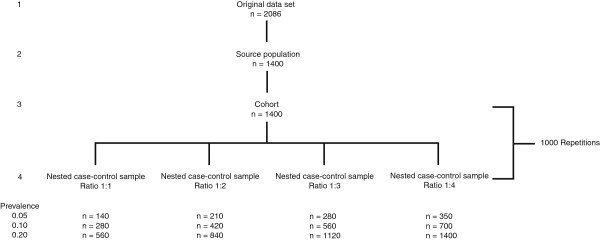
**Flow-chart of the sampling process of the nested case–control samples.** This process was repeated for a deep venous thrombosis prevalence in the source population of 0.05, 0.1 and 0.2. “n” in the nested case–control sample represents the average sample (cases plus controls) size across 1000 samples.

We first studied a source population based on the original data set (Figure 2, line 1), with a prevalence of DVT of 0.1 (140 cases, 1260 controls), reflecting a relatively rare disease situation that commonly directs case–control studies (Figure 2, line 2). The diagnostic accuracy parameters estimated for this source population serve as the commonly unknown true parameter values (see below and Table 1). Subsequently, we mimicked a cross-sectional cohort study of the same size as the source population, i.e. 1400 patients that were drawn with replacement from our source population (cohort, Figure 2, line 3).

**Table 1:** Estimates of diagnostic accuracy of the D-dimer test and the calf-difference test in the source population for different values of the prevalence of deep venous thrombosis (DVT)

**Prevalence of DVT**	PPV*	NPV*	**Sensitivity**	**Specificity**	**DOR**	**Positive test result (%)**
D-dimer test (dichotomous)						
0.05	0.07	0.99	0.96	0.32	10.7	69
0.1	0.14	0.99	0.97	0.33	9.2	70
0.2	0.26	0.97	0.96	0.33	11.9	73
Calf-difference test (dichotomous)						
0.05	0.09	0.98	0.69	0.65	4.0	37
0.1	0.17	0.95	0.68	0.64	3.1	39
0.2	0.32	0.89	0.67	0.65	3.8	41

* Estimates of PPV and NPV obtained with the weighted formula, with Mercaldo’s approach and with weighted logistic regression were the same

(PPV = positive predictive value; NPV = negative predictive value, DOR=Diagnostic Odds Ratio).

A nested case–control sample was then created from the cohort (Figure 2, line 4). We included all patients with DVT (cases) from the corresponding cohort in the nested case–control sample, and an equally sized random sample from the subjects without DVT (controls): case–control ratio = 1:1. To prevent too much sampling errors (random variation), we repeated the above approach 1000 times, creating 1000 study cohorts from the source population and hence 1000 nested case–control samples. In the 1000 nested case–control samples we estimated the predictive values of both index tests and their uncertainty (standard error and 95% CI) using the six approaches described below. All this was also done for three other case–control ratios: 2 controls per case (ratio 1:2); 3 controls per case (1:3); and 4 controls per case (1:4). The prevalence of the 1000 cohorts was thus not fixed across the different cohorts, though with a mean prevalence of 0.1 (95% CI 0.08-0.12). The actual prevalence of the corresponding cohort was used for all subsequent calculations in the nested case–control sample.

Finally, the entire process of creating the 1000 study cohorts and 1000 corresponding nested-case control samples (with the four different case–control ratios), was repeated for a source population (n=1400) with a DVT prevalence of 0.05 (70 cases) and 0.2 (280 cases).

### Approaches to estimate the uncertainty of predictive values of a diagnostic test from a nested case–control study

We compared six approaches to estimate the 95% CI of the predictive values obtained from the nested case–control samples, for the two index tests (D-dimer test and calf circumference difference). The point estimates of the predictive values were obviously the same for all six approaches, while the standard error estimates and hence 95% CI could vary. We describe the approaches for the predictive value of a positive result (positive predictive value = PPV). They can mutatis mutandis be applied to the negative predictive value (NPV). Notations used below, refer to those used in Figure 1 (see legend of Figure 1 for explanation of variable names).

1. Estimate the standard error of the PPV (SE(PPV)) using the standard formula for the SE of a proportion with the actually observed number of patients in the nested case–control sample:

$$\text{SE}\left(\text{PPV}\right)=\sqrt{\frac{\frac{a}{a+b_{1}}\cdot \frac{b_{1}}{a+b_{1}}}{a+b_{1}}}$$

The 95% confidence interval can simply be calculated as PPV ± 1.96*SE(PPV)Calculating the SE with the actually observed numbers in the nested case–control samples (i.e. without correction for the sampling fraction that is used to estimate the correct PPV, using the upweighting by the samping fraction as shown in Table 1), agrees to the number of patients actually measured. However, the proportions in approach (1) do not correspond to the e stimated (corrected) PPV.

2. Estimate the SE(PPV) using the standard formula for SE of a proportion with correction for the sampling fraction in the numerator of approach 1 above, but not in the denominator:

$$\text{SE}\left(\text{PPV}\right)=\sqrt{\frac{\frac{a}{a+\frac{1}{\text{Sampling}\;\text{fraction}}\cdot b_{1}}\cdot \frac{\frac{1}{\text{Sampling}\;\text{fraction}}\cdot b_{1}}{a+\frac{1}{\text{Sampling}\;\text{fraction}}\cdot b_{1}}}{a+b_{1}}}$$

The correction is only applied to the numerator as this reflects the (corrected) PPV estimates. Applying the correction also to the denominator, would make the SE incorrectly too small: a larger number of patients than actually observed would then be used in the SE estimation.

3. Assess the empirical distribution of the PPV using a bootstrap procedure. Per nested case–control sample we drew 1000 bootstrap samples and estimated the PPV in each bootstrap sample. The PPV values corresponding to the 2.5 and 97.5 percentiles of the 1000 bootstrap estimates were used as the limits of the 95% confidence interval.

4. The approach recently described by Mercaldo and colleagues [12]. This approach uses the prevalence from the underlying study cohort (not to be confused with our ‘true’ source population, see above) and the sensitivity and specificity estimated from the case–control sample to calculate the correct PPV. Not only the PPV can be estimated using the sensitivity (Sens) , specificity (Spec) and prevalence (p), but also the SE(PPV):

$$SE\left(PPV\right)=\frac{{\left[p\cdot \left(1-Spec\right)\cdot \left(1-p\right)\right]}^{2}\cdot \frac{Sens\cdot \left(1-Sens\right)}{a+c}+{\left[p\cdot Sens\cdot \left(1-p\right)\right]}^{2}\cdot \frac{Spec\cdot \left(1-Spec\right)}{b_{1}+d_{1}}}{{\left[Sens\cdot p+\left(1-Spec\right)\cdot \left(1-p\right)\right]}^{4}}$$

5. Weighted logistic regression. This is an ordinary logistic regression model with outcome disease present (y/n) and one covariable (index test result, positive or negative), with weights for cases and controls. The model can be written as log odds(PPV) = $\log\left(\frac{ppv}{1-ppv}\right)$ = *α* + *β* ×. With × =1 for a positive index test result. Each case receives a weight w(cases) = $\frac{N_{1}}{N}$ (rather than simply weight 1) and each non-case receives weight w(non-cases) = $\frac{N_{1}}{N}\cdot \frac{1}{\text{Sampling}\;\text{fraction}}$. Hence, the sum of the weights over all sampled subjects equals the total number of subjects in the nested case–control sample (N_1_). This sum equals the effective sample size in the estimations of the PPV and SE(PPV). Results of the so weighted regression analysis are the intercept (α) and the regression coefficient for the index test (β). The standard error of the logit(PPV) can be calculated from the covariance matrix SE(logit[PPV]) = $\sqrt{\mathrm{var}iance\left(\alpha \right)}+\mathrm{var}iance\left(\beta \right)+2\cdot \mathrm{cov}ar\left(\alpha ,\beta \right)$ The covariance matrix is estimated with the correct number of observed (N1) patients, since case and controls were weighted in the analysis.

6. Use the approach by Mercaldo and colleagues (approach 3) [12] on the log odds scale. One uses the sensitivity (Sens) , specificity (Spec) and prevalence (p) in the known study cohort, to estimate the SE of the logit(PPV) by:

$$\text{SE}(\text{logit}\left(\text{ppv}\right)=\left(\frac{1-sens}{sens}\right)\cdot \frac{1}{a+c}+\left(\frac{spec}{1-spec}\right)\cdot \frac{1}{b_{1}+d_{1}}$$

### Statistical analysis

The PPVs of both index tests were thus calculated using the weighting approach from Figure 1. We then estimated the 95% confidence interval of the PPV using the six approaches above. From the 1000 nested case–control samples, the average 95% confidence interval width and the coverage probability were estimated. The narrower the average confidence interval width, the more precise the estimated predictive value[16]. The coverage probability is the proportion of the 1000 confidence intervals that included the true PPV estimated from of the source population. The coverage should not fall outside two SE’s of the nominal probability (p) [16]. Nominal p is 0.05 for a 95% confidence interval, with SE(nominal p) = 0.0069 for a simulation study with 1000 repetitions (Se(p) = $\sqrt{\frac{p\left(1-p\right)}{B}}$, with *B* the number of repetitions). The corresponding coverage ranges from 0.936 – 0.964. If the coverage probability of the PPV’s falls outside this interval we speak of “substantial undercoverage” for lower coverage probability (<0.936), or overcoverage for higher (>0.964) coverage probability.

The ideal estimation approach has a coverage close to 95% and a small 95% confidence interval of the estimated predictive values.

All analyses were executed for the four case–control ratios, and for the three different disease prevalence’s in the source population.

Analyses were performed with R version 2.6.0 [17].

## Results

Table 1 shows the accuracy estimates of both index tests as estimated from the source population. The PPV of both tests was low and the NPV of both tests was high as a result of the low prevalence of DVT. For both tests, the PPV increased and NPV decreased with increasing prevalence of DVT. The D-dimer test was very sensitive with limited specificity. The calf difference test was moderately sensitive and specific. The D-dimer test was positive in 978 (70%) patients for a DVT prevalence of 0.1. The calf-difference test was positive in 568 (41%) patients. Changing the prevalence of diseases did not change the percentage of positive tests. As expected, for both tests, the sensitivity, specificity and diagnostic odds ratio were similar for each prevalence. The point estimate for the PPV and NPV obtained with weighted logistic regression were similar (respectively 0.14 and 0.99) to those obtained with the standard approach.

**Figure 3:**
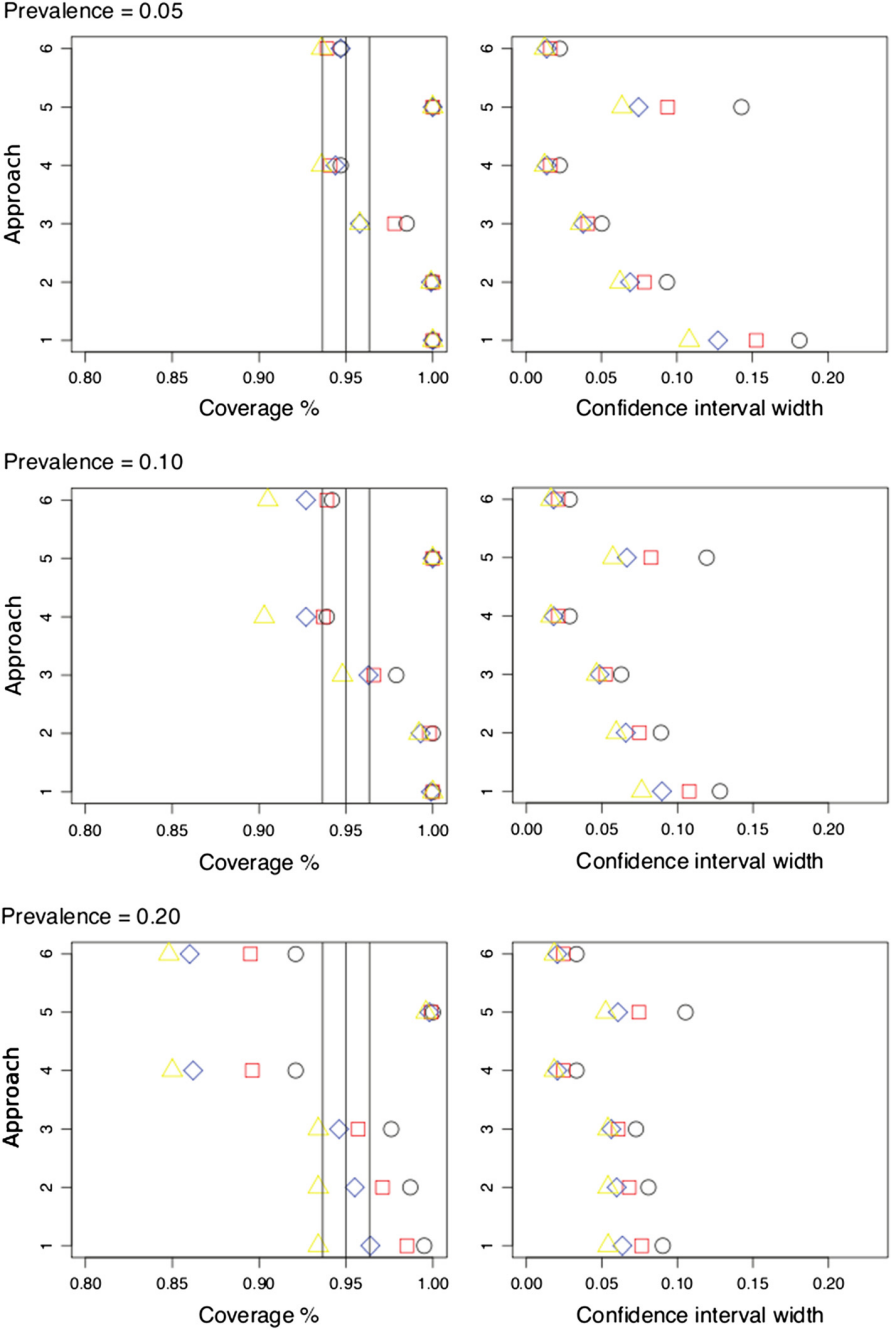
**For each estimation approach (for details see text) and per deep venous thrombosis prevalence, the plot of positive predictive value coverage probabilities and 95% confidence interval width per approach for different prevalence’s for theD-dimer test(1 = Standard formula for obtaining a standard error of a proportion, 2 = as 1**^**st**^**approach but with correction for sampling fraction, 3 = Bootstrap procedure, 4 = Mercaldo and colleagues approach, 5 = Weighted logistic regression, 6 = Logit transformation of Mercaldo and colleagues approach).** Colors/figures represent the different sampling fractions (Black circle = 1 case: 1 control, Red square = 1:2, Blue diamond = 1:3, Yellow triangle = 1:4). The vertical lines represent the ideal 95% coverage with its confidence interval, i.e. the levels of acceptability.

**Figure 4:**
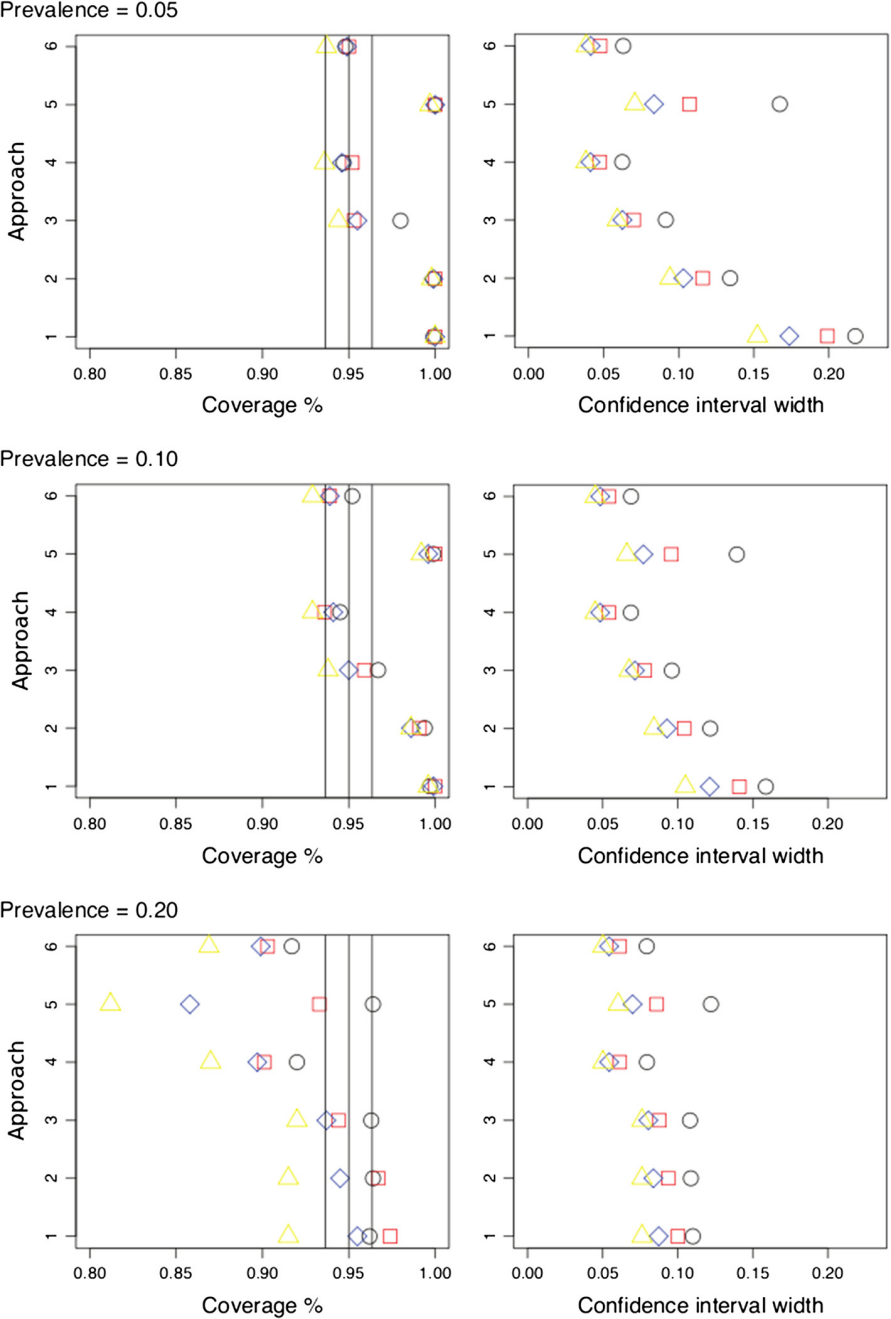
**For each estimation approach (for details see text) and per deep venous thrombosis prevalence, the plot of the positive predictive value coverage probabilities and the 95% confidence interval width, for thecalf difference test.** 1 = Standard formula for obtaining a standard error of a proportion, 2 = as 1^st^ approach but with correction for sampling fraction, 3 = Bootstrap procedure, 4 = Mercaldo and colleagues approach, 5 = Weighted logistic regression, 6 = Logit transformation of Mercaldo and colleagues approach). Colors/figures represent the different sampling fractions (Black circle = 1 case: 1 control, Red square = 1:2, Blue diamond = 1:3, Yellow triangle = 1:4). The vertical lines represent the ideal 95% coverage with its confidence interval, i.e. the levels of acceptability.

Approaches one, two and five showed clear overcoverage at low prevalences of 0.05 and 0.1 in the cohorts for all case–control ratios. They showed less overcoverage at a prevalence of 0.20 and even an undercoverage (Figure 3 and 4, approach 5). Approach three yielded slight overcoverage for lower case–control ratios (1:1, 1:2) and for low prevalences (0.05 and 0.01). Approaches four and six showed undercoverage for higher case–control ratios (1:3, 1:4). Extreme undercoverage was seen at a prevalence of 0.20 (Figure 3 and 4, left panels) for both approach four and six.

In general, approach one showed the largest confidence interval width corresponding to the overcoverage, whereas approach four and six showed very similar and small widths. Approach three showed slightly larger widths then approach four and six (Figure 3 and 4, right panels).

## Discussion

We compared six approaches for estimating the confidence intervals of predictive values or post-test probabilities of diagnostic test results when a nested case–control design is used. using simulations in a large empirical diagnostic study, the six approaches were compared in terms of coverage and the width of the 95% confidence intervals. Our data show that a bootstrap procedure (approach 3) seems to be the preferred approach, although it was only slightly better than the other approaches. Approaches 4 and 6 showed some undercoverage, particularly for the D-dimer test with frequent positive results (positive results around 70%). Approaches 1, 2 and 5 showed overcoverage. For a prevalence of 0.2 in the underlying cohort and a case–control ratio of 1:4 all approaches showed substantial undercoverage. In fact a case–control ratio of 1:4 implies a prevalence of 0.2 in the nested case–control sample. Hence, one may argue that a full cohort study is to be preferred, when the disease prevalence in the cohort is 0.2 or higher. Indeed, case–control studies are notably advantageous when the prevalence of a disease in the cohort is rare (i.e. below 0.1).

By applying a nested case–control design in diagnostic accuracy studies the number of patients undergoing the index test can be substantially reduced, hereby increasing the efficiency of the particular study [6,8,10,11]. This becomes more important if the index test comes with large patient burden, is costly, the disease is rare, and when stored biological material is used for measuring new tests, e.g. from proteomics, metabolomics or genomics. Previously it has been shown that by applying a correction for the sampling fraction precise point estimates of the predictive values can be obtained [5]. We found that applying a bootstrap procedure to estimate the confidence intervals around these predictive values, yields adequate results for the uncertainty in the estimated predictive values. Limitation of this approach can be that, due to the low numbers, in some of the bootstrap samples one of the cells of the 2×2 table remains empty, The latter did not happened in our simulation. If this happens PPV may be estimated with a continuity correction for low numbers.

The predictive values obtained with the approach recently discussed by Mercaldo and colleagues were equal to those derived with the weighted approach from Figure 1. For the lower prevalence’s (0.05 and 0.10) the coverage of approaches 4 and 6 was between 0.90 and 0.95 which were similar to those found by Mercaldo and colleagues themselves [12]. With increasing case–control ratio and increasing prevalence, the Mercaldo and colleagues approach yielded more undercoverage. This could be due to the fact that in their original paper the case–control ratio was not explicitly varied, although in their equation the case–control ratio implicitly has influence on the SE and hence the confidence interval. Besides the study by Mercaldo and colleagues we are not aware of any other studies coping with this issue of uncertainty of predictive values estimated from nested case control studies.

A limitation of our study could be that we looked at only one original cohort in our simulations and studied only two index tests. Although the results for the different combinations simulated are alike, it is thinkable that for other combinations of disease prevalence, cohort sizes, and diagnostic accuracy of the index tests, the results could slightly differ. We certainly realize that DVT is not a true rare disease and most diagnostic studies on DVT are done on a full-cohort and not on a nested case–control sample. Therefore, we slightly modified the prevalence in the full cohort to better mimick the rare-disease situation, which we needed for our comparisons.

By using a fixed cohort size (n=1400) for the different prevalence’s, the size of the nested case–control samples varied (Figure 2). This could have influenced our results slightly since the SE and the confidence interval depends on the number of observations. Alternatively one could use a fixed number of cases in with varying cohort sizes for different prevalence’s.

## Conclusion

Our case-study suggests that in diagnostic accuracy studies using a nested case–control design, one can apply a simple bootstrap procedure to obtain a confidence interval for the post-test probabilities or predictive values of the index test results. For our data-set, the bootstrap procedure showed the best combination of coverage and 95% confidence interval width, compared with the other approaches. Our findings and inferences can also be applied to nested case control studies that investigate the predictive values of results from other kind of tests, for example prognostic tests.

## Competing interests

The authors declare that they have no competing interests.

## Authors' contributions

BvZ designed the study, performed the analysis and drafted the manuscript. YV designed the study and drafted the manuscript. ART Donders designed the study and drafted the manuscript. KGMM designed the study and drafted the manuscript. All authors read and approved the final manuscript.

## Funding

The study was supported by grants of ZonMw, the Netherlands organization for health research and development (project numbers 945-27-009, 918-10-615 and 912-08-004).

## Pre-publication history

The pre-publication history for this paper can be accessed here:

http://www.biomedcentral.com/1471-2288/12/166/prepub
